# Hash Encoding and Brightness Correction in 3D Industrial and Environmental Reconstruction of Tidal Flat Neural Radiation

**DOI:** 10.3390/s24051451

**Published:** 2024-02-23

**Authors:** Huilin Ge, Biao Wang, Zhiyu Zhu, Jin Zhu, Nan Zhou

**Affiliations:** Ocean College, Jiangsu University of Science and Technology, Zhenjiang 212100, China

**Keywords:** industrial diagnostics, 3D reconstruction, image enhancement

## Abstract

We present an innovative approach to mitigating brightness variations in the unmanned aerial vehicle (UAV)-based 3D reconstruction of tidal flat environments, emphasizing industrial applications. Our work focuses on enhancing the accuracy and efficiency of neural radiance fields (NeRF) for 3D scene synthesis. We introduce a novel luminance correction technique to address challenging illumination conditions, employing a convolutional neural network (CNN) for image enhancement in cases of overexposure and underexposure. Additionally, we propose a hash encoding method to optimize the spatial position encoding efficiency of NeRF. The efficacy of our method is validated using diverse datasets, including a custom tidal flat dataset and the Mip-NeRF 360 dataset, demonstrating superior performance across various lighting scenarios.

## 1. Introduction

Unmanned aerial vehicles (UAVs) have become indispensable tools for capturing high-resolution images of tidal flat environments, facilitating detailed 3D reconstructions crucial for the geosciences. Unmanned aerial vehicles (UAVs), renowned for their agility, versatility, speed, and cost-effectiveness, serve as invaluable platforms for aerial photography, enabling the swift capture of high-resolution images with vast potential for generating geographic mapping data [1,2]. Over recent decades, UAV photogrammetry has found applications across various disciplines within the geosciences, including sedimentology [3,4], earthquake geology [5,6], structural geology [7], geomorphology [8], engineering geology [9,10], archaeology [11,12], forestry [13,14], and landscape evolution and natural hazards [15,16]. Innovative techniques such as RTK or PPK aero photogrammetric surveying have yielded numerous accurate 3D models [7,17]. With advancements in photogrammetry, methods for generating dense point clouds and constructing 3D triangular grid models from 2D images have evolved, incorporating sparse reconstruction (structure from motion, SFM) [18,19] and dense reconstruction (multiple-view stereo, MVS) [20,21]. This progress has effectively transformed the reconstruction of 3D building models into a practical reality. However, during the UAV surveys, challenges arise from brightness variations caused by factors like sunlight exposure and water surface reflections. This article addresses these challenges by proposing advanced techniques within the neural radiance fields (NeRF) [22] framework for UAV-based 3D reconstruction in both industrial and environmental contexts.

This introduction provides an overview of related work, emphasizing NeRF’s significance in 3D reconstruction and highlighting ongoing efforts to enhance its efficiency and performance in dynamic and challenging environments. The research gap is identified regarding NeRF’s handling of brightness conditions in tidal flats, particularly within industrial applications [23].

The proposed methodology employs a two-fold strategy. First, we introduce a hash encoding technique to optimize the encoding of spatial positions and input perspectives in NeRF, enhancing its efficiency for industrial diagnostics. Second, a novel luminance correction method is presented, integrating a convolutional neural network (CNN) to address common overexposure and underexposure issues in UAV-captured images of tidal flats. The goal is to enhance the accuracy and realism of 3D reconstructions, making them adaptable to diverse lighting conditions in both industrial and environmental settings.

To evaluate the effectiveness of the proposed methods, experiments are conducted using both a custom tidal flat dataset and the Mip-NeRF 360 dataset. Performance metrics, including PSNR, SSIM, and LPIPS, are employed to assess the quality and similarity of synthesized images. The results demonstrate the superiority of the proposed approach in achieving more accurate reconstructions, particularly in challenging industrial environments with varying luminance conditions.

In conclusion, this article makes a significant contribution to the field of UAV-based 3D reconstruction for industrial diagnostics by specifically tackling the challenges presented by brightness variations in tidal flat environments. The introduced techniques, encompassing hash encoding and luminance correction, augment the capabilities of NeRF, offering a more resilient solution for authentic 3D scene synthesis in both industrial and environmental applications. The ramifications of this work extend to industries and researchers engaged in environmental monitoring and industrial diagnostics.

## 2. Related Work

### 2.1. 3D Reconstruction with NeRF

Neural radiance fields (NeRF) seamlessly integrate classical computer graphics concepts with machine learning techniques to generate images derived from real-world observations normally performed with optical sensors or imaging devices [24]. This innovative approach utilizes a fully connected deep neural network to model scenes, combining it with traditional volume rendering methods to project calculated colors and densities into an image. This methodology surpasses prior techniques in terms of image quality, allowing for the rendering of high-resolution, photorealistic views.

The widespread recognition and potential of NeRF underscore the necessity for continuous refinement of this algorithm. Current research is focused on enhancing the NeRF algorithm through various innovative methods. A primary focus is on augmenting the training and inference efficiency of the network. Considering the substantial time and computational resources required for training NeRF models, improvements in efficiency are essential for their viability in practical applications. In their work, Lindell et al. [25] propose automatic integration, presenting a novel framework for acquiring efficient, closed-form solutions to integrals through the use of coordinate-based neural networks. Additionally, DONeRF [26] introduces a compact dual network design. In contrast to concurrent acceleration methods for ray-marching-based neural representations, DONeRF does not necessitate additional memory for explicit caching or acceleration structures. Moreover, it can achieve interactive rendering at 20 frames per second on a single GPU.

Furthermore, there are ongoing efforts to enhance the performance of NeRF models in processing dynamic scenes and irregular surfaces. Traditional NeRF methodologies may encounter difficulties under these conditions due to their dependence on static scenes and high-quality data inputs. Barron et al. proposed a solution named “Mip-NeRF [27]”, which reduces objectionable aliasing artifacts and significantly improves NeRF’s ability to represent fine details. To improve few-shot quality, Jain et al. proposed DietNeRF [28], which introduce an auxiliary semantic consistency loss that encourages realistic renderings at novel poses. DS-NeRF [29] can render better images given fewer training views while training 2–3x faster.

In addition, NeRF is explored to further refine the model’s accuracy and robustness in varied and challenging real-world environments, such as cases of reflection [30], noise [31], blur [32], underwater environments [33], and glossy surfaces [34]. At present, there is also work to improve the performance of NeRF under different brightness conditions. NeRV [35] present a method that introduces surface normal and material parameters as output. NeRF-OSR [36] enables direct control over the scene illumination, as defined through a spherical harmonics [37] model. However, there has been little work to optimize the light condition of the tidal flat environment.

### 2.2. NeRF Encoding Method

Encoding inputs in higher-dimensional spaces undoubtedly confers substantial advantages in neural graphics and machine learning models [38]. This strategy empowers the model to discern intricate patterns that might elude detection in lower-dimensional spaces. Early examples, including one-hot encoding [39] and the kernel trick [40], set the stage for more sophisticated techniques. In recent research, these input encodings have played a pivotal role in augmenting the attention components of recurrent architectures [41]. The advent of transformers [42] by Vaswani et al. has further broadened the capabilities of neural networks, particularly in pinpointing processing locations within the data.

Many new advances have been made in parameter encoding. Chabra et al. introduced Deep Local Shapes [43] (DeepLS), a deep shape representation that enables encoding and reconstruction of high-quality 3D shapes without prohibitive memory requirements. Jiang et al. train an autoencoder to learn an embedding of local crops of 3D shapes at that size. Liu et al. introduce neural sparse voxel fields [44] (NSVF), a new neural scene representation for fast and high-quality free-viewpoint rendering.

This evolution of input encoding methodologies has had a profound impact on the field of computer graphics, especially with the advent of NeRF by Mildenhall et al. [22]. NeRF’s innovation is rooted in its efficient encoding of spatial positions and input perspective directions. The breakthroughs in NeRF, largely credited to these advanced encoding techniques, have empowered more accurate and realistic 3D scene reconstructions from 2D images. In the context of NeRF, the encoding of inputs into higher-dimensional spaces is especially crucial for handling the complexity of light and density fields in 3D environments. These encodings assist in accurately capturing the subtleties of light interactions and spatial relationships, which are vital for realistic renderings [45]. Müller et al. introduce a multi-resolution hash encoding [46] technique for the real-time rendering of neural graphics primitive. This method significantly enhances the efficiency of NeRF models by optimizing data structures and retrieval processes. Our decision to adopt hash coding instead of using the sine and cosine functions in NeRF was inspired by this innovative approach.

### 2.3. Improvement under Demanding Lighting Circumstances

When acquiring images of tidal flats through UAVs, the extended time span of acquisition leads to a substantial brightness disparity between images taken in the morning and at night. This contrast is predominantly influenced by various factors, including distinct light source exposures, the characteristics of light reflected from the water surface, and variations in camera exposure time. The interplay of these intricate optical effects collectively impacts the quality and brightness of the obtained images, subsequently influencing the accuracy and reliability of subsequent image processing and analysis. To address these challenges, multiple techniques for image enhancement and exposure correction have been developed and proposed.

Traditional methods are mainly used to process images with poor lighting conditions, including RetiNex theory [47] or histogram equalization [48]. The mainstream solutions is based on deep neural networks (DNNs) methods. Wei et al. introduced an enhancement network called Enhance-Net [49] for subsequent lightness enhancement of illumination following decomposition, and a denoising operation is applied to the reflectance for joint denoising. DeepLPF [50] introduces a deep neural network named Deep Local Parametric Filters, which regresses the parameters of spatially localized filters that are subsequently automatically applied to enhance the image. EnlightenGAN [51] is presented as a highly efficient unsupervised generative adversarial network that can be trained without the need for low/normal-light image pairs. At the same time, image exposure is critical to image quality. Afifi et al. address this problem by proposing a deep neural network (DNN) model that is trained from coarse-to-fine in an end-to-end manner [52]. Nsampi et al. utilizes a global attention mechanism that allows for distant interaction between distant pixels for exposure correction [53]. Cui et al. proposed a lightweight and fast IAT for recovering normal sRGB images from low-light and underexposed or over-exposed conditions by performing local and global image decomposition of the image signal processor (ISP) pipeline [54]. Huang et al. leveraged the relationship information between images with different exposure levels in a small batch as an important constraint during network training to improve the optimization consistency of the exposure correction model [55].

However, existing methods addressing image enhancement primarily concentrate on optimizing images rather than on generating coherent 3D scenes for new views. To tackle this issue, we introduce a convolutional neural network [56] to extract luminance features from images. A loss function for unsupervised luminance correction is incorporated into NeRF, enhancing new view synthesis and improvement under low-light and overexposure conditions.

## 3. Methods

### 3.1. Neural Radiance Field

Neural radiance fields (NeRF) were introduced as a groundbreaking method for synthesizing 3D scenes through the application of deep learning, signifying a significant advancement in computer graphics and 3D modeling. NeRF utilizes a fully connected deep neural network to map 5D coordinates (spatial XYZ and 2D viewing directions) to color and volume density. However, neural networks encounter challenges in learning high-frequency information [57]. To address this issue, NeRF encodes the input data using sine and cosine functions, allowing for a better fit to data with high-frequency variations, as demonstrated in Formula (1):

(1) $$\gamma^{k}:p\to \left(\begin{array}{c} \sin\left(2^{0}p\right),\cos\left(2^{0}p\right),\ldots ,\sin\left(2^{k}p\right),\cos\left(2^{k}p\right) \end{array}\right).$$

The radiation field can be conceptualized as a function where the input is a ray in space **r**(*t*) = **o** + *t*·**d** (**r** ∈ **R**). We can use this function to query the density σ of the ray **r**(*t*) at each point (*x*, *y*, *z*) in space, as well as the color **C**(**r**) that is rendered in the direction **d** of the ray. The density *σ* also signifies the probability value that a ray will terminate at this point in space and controls the amount of radiation absorbed by other rays as they pass through the point.

When drawing an image for a given position o and direction d, the radiation from all points on a given ray **r**(*t*) is accumulated to compute the color value **C**(**r**) of the corresponding point in the image. Formally,

(2) $$C\left(r\right)=\int_{t_{n}}^{t_{f}}T\left(r\left(t\right)\right)\sigma \left(r\left(t\right)\right)c\left(r\left(t\right),d\right)dt,$$

(3) $$T\left(r(t)\right)=exp\left(-\int_{t_{n}}^{t}\sigma \left(r(s)\right)ds\right).$$

In Formula (2), time is denoted as “*t*”, with $t_{n}$ and $t_{f}$ representing the start and end points. The rendering outcome is derived from the interplay of three critical factors: the cumulative transmittance $T\left(r\left(t\right)\right)$, the density $\sigma \left(r\left(t\right)\right)$, and the color $c\left(r\left(t\right),d\right)$. Crucially, the interaction of cumulative transmittance $T\left(r\left(t\right)\right)$ and density $\sigma \left(r\left(t\right)\right)$ serves as a “color weight” parameter for a given spatial point. This parameter essentially quantifies the remaining light intensity at a specific point and its corresponding density. As depicted in Formula (3), this relationship follows an inverse exponential pattern. This implies that a higher density at a given point results in a proportionately lesser amount of light penetrating beyond that point.

In the actual rendering process, the discrete form of Formulae (1) and (2) are represented as follows:

(4) $$C\left(r\right)=\sum_{i=1}^{N}T(r(i))(1-exp(-\sigma (r(i))\cdot \delta_{i}))\cdot c(r(i),d),$$

(5) $$T(r(i))=exp(-\sum_{j=1}^{i-1}\sigma \left(r(j))\cdot \delta_{i}\right).$$

In Formula (4), $\delta_{i}=t_{i+1}-t_{i}$, the relation between $\sigma \left(r\left(t\right)\right)$ and $\left(1-exp\left(-\sigma \left(r\left(i\right)\right)\;\cdot \delta_{i}\right)\right)$ has been proved [58].

NeRF focuses solely on objects in space and not on empty areas. However, since empty spaces constitute the majority of the space, the rendering method based on uniform random ray sampling is less efficient in this case. The rendering process of NeRF involves a weighted summation of the colors of the sampled points on the light, as depicted in Formula (4), where the weight $w_{i}=T(r(i))(1-exp(-\sigma (r(i))\cdot \delta_{i}))$. Two neural networks are trained, a coarse network $F_{\sigma }$ and a fine network $F_{c}$, specifically,

(6) $$F_{\sigma }(r(i))\to \sigma (r(i)),h,$$

(7) $$F_{c}(h,d)\to c(r(i),d),$$

where **h** is the feature vector sent by the coarse network $F_{\sigma }$ to the fine network. The Sigmoid and RELU activation functions are used to normalize the range of values of color c(r(i),d) and density σ(r(i)) to [0, 1] and [0, ∞].

A set of rays is sampled at N_coarse_ and the coarse network is evaluated at these locations. Combining the results of this coarse network, the output of N_fine_ locations is sampled from this distribution using inverse transform sampling, and the data from the N_coarse_ and N_fine_ sampling are then fed into the fine network and the final rendered light color **C**®. The optimization of NeRF involves minimizing the mean square error loss between the predicted image $\hat{C}\left(r\right)$ and the ground truth image C(r), specifically,

(8) $$L_{mse}=\sum_{r}^{R}||\hat{C}(r)-C(r)|{|}^{2}.$$

### 3.2. Hash Encoding

In practical applications, describing an object’s contours often requires a limited set of parameters. However, a substantial number of parameters are typically allocated to define relatively small surface regions that might have a lesser impact on the overall model performance. Unfortunately, this results in increased computational demands for processing and storing these parameters.

In the NeRF approach, as demonstrated in Formulae (6) and (7), two multi-layer perceptrons (MLPs) are trained to model features within the target scene. Despite its effectiveness, NeRF requires updating the entire weights of the MLP during each training iteration. To alleviate unnecessary computational overhead without compromising reconstruction quality, we propose partitioning the target 3D space into cubes of varying sizes. By interpolating the eight vertices of each cube, we efficiently capture information about the points inside it. Consequently, only the scene features corresponding to the eight cube vertices need updating in each training cycle, significantly reducing the computational burden.

**Figure 1:**
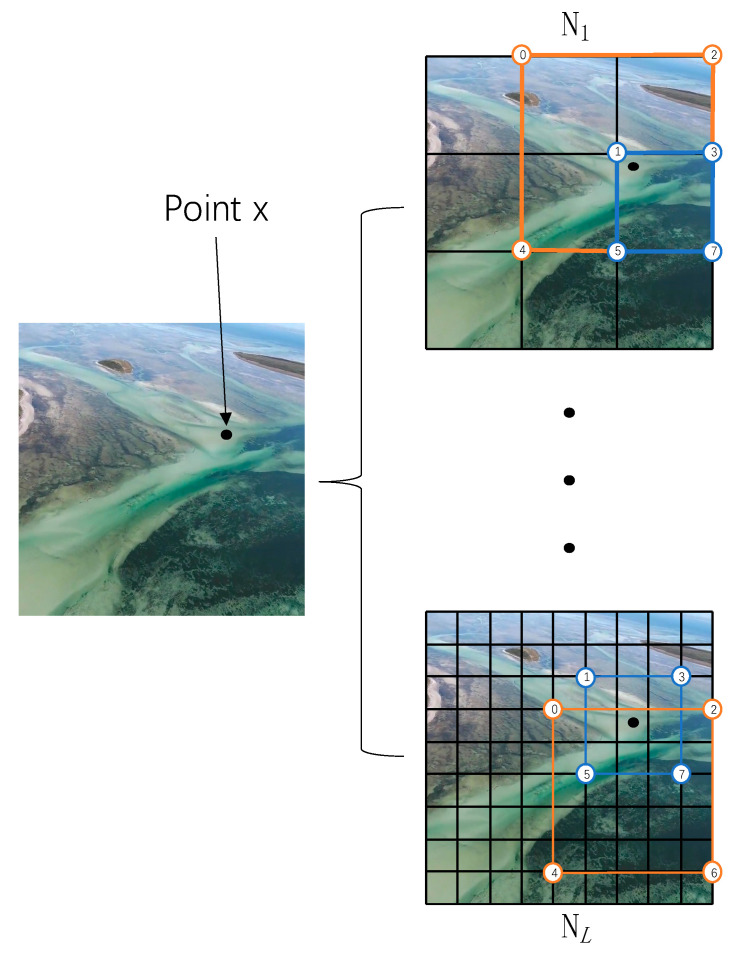
Feature cube in 2D representation.

The entire space are arranged into L levels, and each level corresponds to a cube vertex containing two feature vectors. As shown in Figure 1, we represent it in the form of a plane for ease of expression, and the cubes correspond to the grids in the figure. Using the vertices of different rectangles to represent the vertices of the cube, represented by number 0–7. The resolution relationship between different grids is defined by setting the maximum grid resolution $N_{max}$ and the minimum grid resolution $N_{min}$:

(9) $$b=(N_{max}/N_{min}{)}^{1/(L-1)}.$$

The resolution of the Lth grid is as follows:

(10) $$N_{l}={\lfloor N}_{min}\cdot b^{l-1}\rfloor .$$

**Figure 2:**
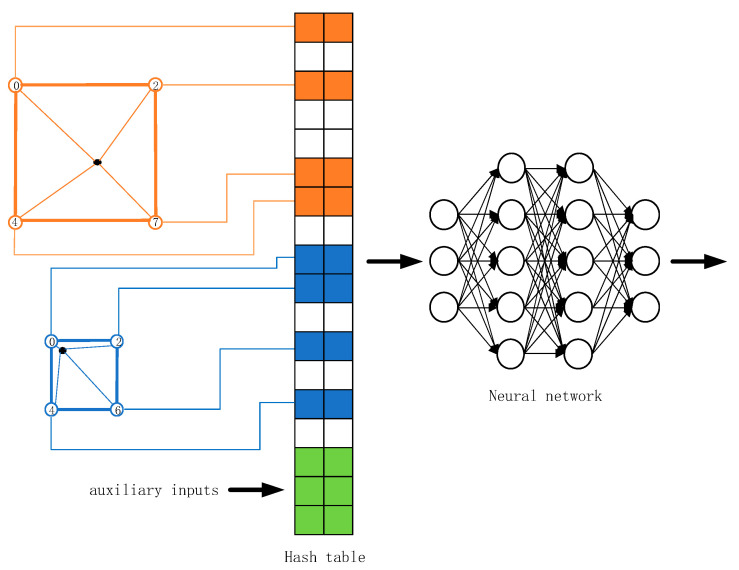
Multi-resolution hash transform.

In Figure 2, given a sampling point ***x*** (*x*_1_, *x*_2_, *x*_3_), various colors are employed to denote the feature cubes at different resolutions. we identify feature cubes of different resolutions containing ***x*** and their corresponding vertex indices at L levels. The feature vectors associated with these vertex indices are retrieved from the hash table of the respective level, where the hash table stores the feature vectors of each vertex, specifically,

(11) $$H(x)=(x_{1}\cdot \pi_{1}⊕x_{2}\cdot \pi_{2}⊕x_{3}\cdot \pi_{3})\mathrm{mod}W.$$

In Formula (11), ⊕ denotes the bit-wise XOR operation, *W* is the hash table’s maximum size, and π is a prime number (π_1_ = 1, π_2_ = 2,654,435,761, and π_3_ = 805,459,861). 

Based on the calculation of the position of *x* in the feature cube, the obtained feature vectors are trilinearly interpolated to compute the feature vector corresponding to the position of ***x***. Subsequently, the feature vectors obtained via interpolation and the observation direction d from the position information are input into the MLP to estimate the density σ and color c of the ***x*** point.

### 3.3. Image Generation for Lighting Challenges

Several factors can contribute to the overexposure and underexposure of images collected by UAVs [59], including uneven lighting conditions due to sunlight exposure, weather changes, and reflections from water surfaces and sandy beaches in tidal flat environments. These factors may result in anomalies in light captured by the sensor. Our objective is to process overexposed and underexposed images to obtain images with normal brightness.

**Figure 3:**
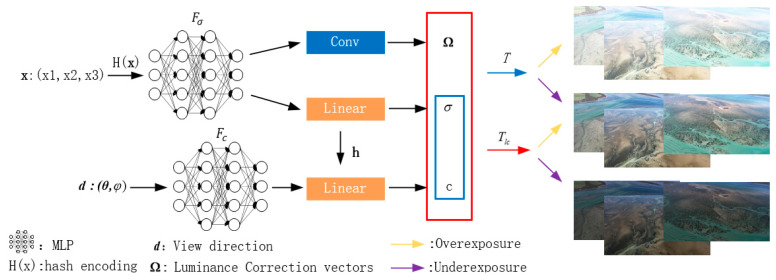
Solutions to tidal flat brightness issues.

In addition to the original NeRF, we rendered an additional set of images to cope with the changing brightness of the tidal flats, as illustrated in Figure 3. The detailed process involves hash-coding the 3D position information of each point (using the encoding method from Formula (1) for viewpoint information) and utilizing this encoded information as the network input. Following the original NeRF method, the density σ corresponding to the spatial point is computed from the position information. To compute the luminance correction vector Ω(r(i)), a convolutional neural network is added (with a size of 7 [60]), specifically,

(12) $$conv(F_{\sigma }(r(i)))\to \Omega (r(i)).$$

This convolution process establishes spatial relationships between pixels and capitalizes on information primarily related to light rather than structure, enough to help the model better understand light and shadow effects. And more pixels are considered when calculating new feature maps. This means that the noise of individual pixels has less impact on the final result, contributing to smoother rendering results.

Luminance correction vector Ω(r(i)) can help us obtain an image with relatively lower brightness than the conventional NeRF, the result of rendering with $T_{lc}$ in Figure 3 as follows:

(13) $$T_{lc}\left(r(i)\right)=exp(-\sum_{j=1}^{i-1}\sigma \left(r(j)\right)\cdot \delta )\cdot \prod_{j=1}^{i-1}\Omega \left(r(j)\right).$$

Settings rendered for both sets of images use the same underlying density field σ(r(t)) along each camera ray **r**(*t*). For the collected overexposure tidal flats images, two sets of images are rendered by using T and $T_{lc}$, respectively, according to the yellow arrows in Figure 3. We calculated the loss function $L_{1}$ between the image rendered by T and the overexposed training image in order to get closer to the true value of the normal image rendered using $T_{lc}$. For the captured underexposed tidal flats images, according to the purple arrows in Figure 3, the loss function $L_{1}$ between the $T_{lc}$ rendered image and the overexposed training image is computed. So, we can get closer to the real value of the normal image rendered with T.

**Figure 4:**
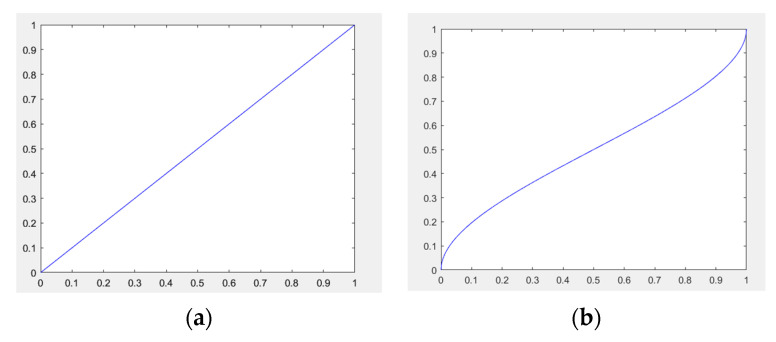
(**a**) $L_{mse}$ loss function curve; (**b**) $L_{1}$ loss function curve.

NeRF optimizes the difference between the rendered image and the real value by calculating $L_{mse}$. However, in the case of overexposure and underexposure images, white and black pixels occupy more weight, and directly using $L_{mse}$ to optimize will result in a brighter or darker rendered image. An inverse tone curve [61] is introduced to rebalance the weights between pixels. The inverse tone curve usually uses a non-linear function, which means it does not treat all pixel values equally. By emphasizing dark details more and compressing highlights, it allows for a more even weighting of pixel values across the tonal range, rebalancing the tonal distribution in the image, denoted as Φ:

(14) $$\begin{array}{c} \begin{array}{c} \Phi (x)=\frac{1}{2}-sin(\frac{sin^{-1}(1-2x)}{3}), \end{array}\\ L_{1}=\sum_{r}^{R}||\Phi (\hat{C}(r)+\varepsilon )-\Phi (C(r)+\varepsilon )|{|}^{2} \end{array}$$

where ε is a constant (defined as 1 × 10^−3^). The comparison of $L_{mse}$ and $L_{1}$ is shown in Figure 4. 

In order to adapt to tidal flat scenes with different luminance, it is possible to control the intensity of the image enhancement by introducing $L_{2}$, as follows:

(15) $$L_{2}=||avgpool(\hat{C}\left(r)\right)-e|{|}^{2}.$$

**Figure 5:**
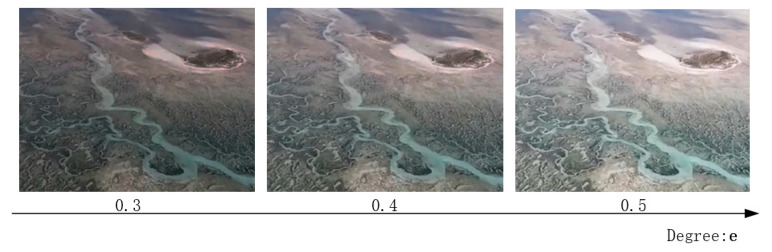
Ablation experiments with constant e.

In this case, $\hat{C}\left(r\right)$ is the color value of the rendered normal image, and **e** is defined as the constant 0.4. Different values of **e** are compared, as shown in Figure 5.

## 4. Experiments

### 4.1. Datasets

In order to verify the performance of our algorithm during overexposure and underexposure, we validate the superiority of our method and the NeRF method with two datasets. We use the Mip-NeRF 360 [62] dataset and the dataset we made by collecting tidal flats environments [63].

The reason for choosing the Mip-NeRF 360 dataset is that the tidal flats environment is a borderless scene. In this environment, the camera field of view may need to capture the full range of the scene, and the Mip-NeRF 360 dataset provides just such panoramic image data.

In the present investigation, we opted for the utilization of a DJI Phantom 4 Pro unmanned aerial vehicle (UAV) for the acquisition of aerial imagery, aiming to procure data of superior quality for our study. Renowned for its exceptional performance and cutting-edge functionalities, the DJI Phantom 4 Pro serves as an unwavering aerial photography platform in the context of this research. The onboard camera of choice is equipped with a 1-inch 24-megapixel CMOS sensor within the Phantom 4 Pro framework. Distinguished by a mechanical shutter and a spacious aperture, this camera is adept at capturing clear and intricate images across varying lighting scenarios.

**Figure 6:**
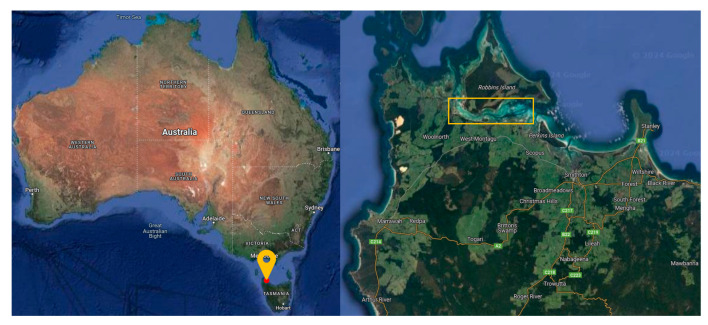
Location map of the survey sample area.

Australia boasts a diverse tidal flat ecosystem, spanning thousands of kilometers of coastline and encompassing various tidal flat types and ecological landscapes. To ascertain the algorithm’s efficacy in a mudflat setting, as shown in Figure 6, we employed an unmanned aerial vehicle (UAV) to capture aerial imagery of tidal flats situated between Smithton and Woolnorth in northwestern Tasmania, Australia, at an altitude of 1000 feet. Subsequently, a comprehensive mudflat dataset was curated, featuring images with a resolution of 1280 × 720. These images were meticulously categorized into distinct scenes, including “tidal tree”, river mouths, ground textures, vegetation, and deep-water areas, with each scene comprising 30–90 images. This dataset serves as a valuable resource for evaluating and validating the algorithm’s performance under tidal flat environmental conditions.

The computational resources harnessed for the execution of our algorithms comprised two NVIDIA GeForce RTX 3090 Ti graphics cards. Similar to conventional NeRF, we use colmap [64] to estimate the camera pose and we also use the Pytorch framework. An adam optimizer is used with an initial learning rate of 5 × 10^−4^, and a cosine learning rate decay strategy is used every 2000 iterations. The training batch size is set to 4096 with a total of 90,000 iterations.

### 4.2. Methodology of the Evaluation

Generating new views is achieved by synthesizing images, so we use PSNR, SSIM, and LPIPS evaluation metrics to comprehensively assess the synthesized images. With these metrics, we are able to synthesize the structural similarity, luminance contrast, and perceptual differences between the synthesized image and the real image to assess the quality and similarity of the newly generated view more comprehensively.

PSNR is a traditional metric for measuring image quality and is calculated based on the peak signal-to-noise ratio of the image, as follows:

(16) $$MSE=\frac{1}{mn}\sum_{i=0}^{m-1}\sum_{j=0}^{n-1}\;|\left|I\left(i,j\right)-K\left(i,j\right)\right|{|}^{2},$$

(17) $$PSNR=10{log}_{10}\left(\frac{{\left(2^{n}-1\right)}^{2}}{MSE}\right).$$

A higher PSNR value indicates superior image quality, serving as a quantitative measure of the similarity between the original and synthesized images by assessing their signal-to-noise ratios. The increased PSNR signifies a closer alignment of structural information between the two images, resulting in elevated overall image quality.

The SSIM is a metric employed to gauge the structural resemblance between two images. SSIM comprehensively considers three critical aspects of information: brightness, contrast, and structure. It evaluates the similarity by scrutinizing the local patterns within the images. SSIM values fall within the range of [−1, 1], and the closer the value is to 1, the greater the resemblance between the two images. SSIM serves as a quantitative measure of the structural similarity between two images, offering insights into the likeness of local patterns within the images.

(18) $$SSIM\left(x,y\right)=\frac{\left(2\mu_{x}\mu_{y}+c_{1}\right)\left(2\sigma_{xy}+c_{2}\right)}{\left({\mu_{x}}^{2}+{\mu_{y}}^{2}+c_{1}\right)\left({\sigma_{x}}^{2}+{\sigma_{y}}^{2}+c_{2}\right)}$$

These include the mean of *x* and *y*$,\;\mu_{x}$ and $\mu_{y}$, the covariance of *x* and *y*,$\;\sigma_{xy}$, the *x* variance, $\sigma_{x}$, and the *y* variance, $\sigma_{y}$, as well as the constants $c_{1}$ and $c_{2}$, which are used to maintain stability.

LPIPS [65] is a deep learning-based image similarity metric that uses artificial intelligence to learn the perceptual differences between images. PIPS not only takes into account pixel-level differences but also focuses on differences perceived by the human eye. It quantifies the perceptual differences between images through the image representations learned by the neural network; the lower the LPIPS value, the more similar the two images are in terms of perception. The process of evaluation is as follows: *x* and *x*0 to be compared are fed into the VGG network or Alexnet, and the output after each layer of the activation function is taken out, normalized, and finally multiplied by the weights to find the error between the two network feature vectors. The error is finally averaged to obtain the final similarity output. The lower the image similarity, the greater the difference between the depth features, the greater the LPIPS output; so, the smaller the LPIPS, the better. LPIPS is learned from deep learning models that can learn perceptual similarity directly from image data without manually designing features. This contributes to the model’s ability to generalize across different tasks and datasets.

### 4.3. Experimental Results

As shown in Table 1, we compare the results of our method and NeRF method on the borderless scenario dataset. It can be seen that on the borderless dataset, our method achieves better experimental results than NeRF on PSNR, SSIM, and LPIPS, indicating that our method is more accurate and more reliable in terms of image reconstruction. The PSNR has increased noticeably by 1.386. Similarly, the SSIM has gone up by 0.058. Moreover, the learned perceptual image patch similarity (LPIPS) has decreased by 0.101.

**Figure 7:**
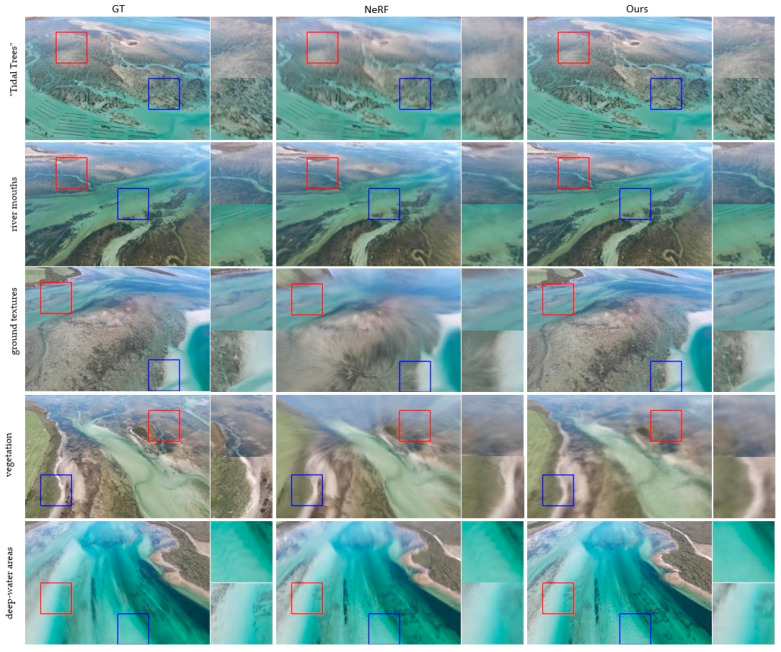
Comparison of renderings of tidal flats environments.

We have similarly compared the performance of the two algorithms in the tidal flats environment, the details of which are shown in Figure 7. It can be seen that under the same tidal flats environment, our method performs better compared to NeRF and more accurately depicts the details of the tidal flats environment.

Figure 7 unmistakably illustrates the superior performance of our method within the same tidal flats environment, showcasing more intricate and nuanced results. In the “ground textures” scene group, the traditional NeRF algorithm produces images with inaccuracies along the edges of the tidal flats. This critical error holds significance for both precise rendering and the preservation of tidal flats environments, potentially leading to avoidable complications.

Through comparative experiments, our method consistently outshines the traditional NeRF algorithm in terms of image quality and accuracy. This observation underscores the enhanced reliability of our method in modeling and rendering tidal flats environments. Our approach adeptly captures scene details, providing a robust tool for both tidal flats research and conservation efforts.

The evaluation metrics presented in Table 2 reveal the differences in image quality and similarity between the two methods, as measured by PSNR, SSIM, and LPIPS values. Our method consistently achieves higher scores across these metrics, affirming its superiority in the challenging tidal flat environment. The PSNR has increased noticeably by 0.501. Similarly, the SSIM has gone up by 0.086. Moreover, the learned perceptual image patch similarity (LPIPS) has decreased by 0.123.

## 5. Discussion

We introduce a novel hash-coding-based approach for the 3D reconstruction of neural radiance fields, specifically tailored for correcting brightness variations in tidal flat environments. Our method involves partitioning the target 3D space into cubes of diverse sizes, facilitating the efficient capture of interior point information. A multi-resolution hash transformation is introduced, employing vertex interpolation to effectively capture interior point details within each cube. This methodology mandates solely updating the scene features corresponding to cube vertices during the training cycle, thereby markedly alleviating the computational load.

To counteract potential overexposure and underexposure issues in images obtained from UAV surveillance, an additional neural network is trained for luminance feature extraction between images, mitigating the impact of disparate exposure conditions on rendered images. An inverse tone curve is incorporated to rebalance pixel weights, ensuring a more harmonious image rendering outcome. Simultaneously, the intensity of image enhancement is modulated by refining the loss function, accommodating diverse brightness levels in tidal flat scenes. These comprehensive advancements collectively contribute to the efficacy of our proposed method in addressing the intricacies associated with brightness correction in the context of 3D reconstruction within tidal flat environments.

However, our algorithm does exhibit certain limitations. First, to address the challenge of brightness variations, we incorporate additional image rendering, thereby augmenting the algorithm’s time overhead. Second, we have not accounted for the influence of meteorological factors on the tidal flats environment, and there are deficiencies in our sampling under relatively adverse weather conditions. Given the sensor’s sensitivity to atmospheric conditions such as smoke and rainfall, the reconstruction quality may be detrimentally impacted.

Consequently, we envisage enhancing the NeRF algorithm in forthcoming work. Our enhancement strategy involves amalgamating lightweight models and introducing implicit coding to regulate environmental factors. Furthermore, we intend to seamlessly integrate denoising and restoration techniques into the preprocessing stage to adeptly contend with intricate weather conditions, thereby substantively augmenting the algorithm’s performance. Further exploration of algorithms in industrial scenarios [66] may be performed in future. This series of enhancements is designed to adeptly address the aforementioned challenges and propel the algorithm towards achieving superior efficacy in real-world applications.

## 6. Conclusions

In summary, this paper presents a novel 3D reconstruction algorithm tailored specifically for the challenges posed by tidal flats environments. Leveraging hash coding techniques, we enhance the extraction of spatial connections from input location information, thereby improving the accuracy of the reconstruction process. Moreover, to address variations in brightness and darkness inherent in UAV-collected tidal flats imagery, we introduce a novel method utilizing CNN networks to extract brightness relationships among the images. This approach effectively mitigates uneven brightness issues encountered during UAV data collection. Furthermore, we propose a new loss function designed to regulate image enhancement strength relative to the NeRF algorithm, as verified through relevant ablation experiments. Our comprehensive evaluation, conducted using both the Mip-NeRF 306 public dataset and our self-collected tidal flats dataset, demonstrates the effectiveness of our algorithm. Through rigorous assessment using three evaluation metrics—PSNR, SSIM, and LPIPS—we validate its superior performance compared to existing methods. By addressing key challenges in 3D reconstruction, our algorithm paves the way for improved understanding and analysis of tidal flats environments, with potential applications in environmental monitoring, coastal management, and beyond.

## Figures and Tables

**Table 1:** Comparison results for the borderless scenario dataset.

	PSNR		SSIM		LPIPS	
Method	NeRF	Ours	NeRF	Ours	NeRF	Ours
Bicycle	23.429	23.075	0.627	0.630	0.404	0.223
Bonai	22.863	25.177	0.549	0.775	0.365	0.383
Counter	22.518	25.557	0.722	0.743	0.320	0.267
Garden	22.520	23.720	0.686	0.701	0.331	0.193
Room	23.318	26.206	0.653	0.841	0.383	0.231
Stump	22.498	22.727	0.688	0.603	0.378	0.282
Average	23.024	24.410	0.654	0.712	0.364	0.263

**Table 2:** Comparison of algorithms for tidal flats environments.

	PSNR		SSIM		LPIPS	
Method	NeRF	Ours	NeRF	Ours	NeRF	Ours
“Tidal Trees”	20.485	20.639	0.523	0.608	0.454	0.230
river mouths	21.475	23.092	0.635	0.784	0.304	0.145
ground textures	21.913	25.231	0.559	0.874	0.451	0.209
vegetation	21.819	21.918	0.576	0.604	0.572	0.589
deep-water areas	24.304	21.620	0.753	0.604	0.391	0.383
Average	21.999	22.500	0.609	0.695	0.434	0.311

## Data Availability

Data are contained within the article.
